# Supplementary material for: Content-rich biological network constructed by mining PubMed abstracts
Source: BMC Bioinformatics. 2004 Oct 8;5:147. doi: 10.1186/1471-2105-5-147 (PMC528731; doi:10.1186/1471-2105-5-147)
Supplement: Additional File 2 — The original results of the above study (non-essential files are deleted to keep the file size under the limit set by BMC bioinformatics). [file 1471-2105-5-147-S2.bz2 › chilibotAdditionalFile2/dip10/503ID10519421E10670/html/TGFB1_TGFBR3.html]

 


 **TGFB1** and **TGFBR3** 
  
Found 81 abstracts in PubMed, retrieved 10.  
 

 What does Google say? 
 PDF only 
| .edu only 

---

**Interactive relationship** (e.g. stimulation, inhibition, etc)

**Inhibitory relationship**- Betaglycan  [ **TGFBR3** ]  inhibits TGF beta  [ **TGFB1** ]  signaling by preventing type I type II receptor complex formation.  Ref: 11668175 J Biol Chem, 2002
- Our data indicate that betaglycan  [ **TGFBR3** ]  can function as a potent inhibitor of TGF beta  [ **TGFB1** ]  signaling by a novel mechanism and provide support for an essential but complex role for proteoglycan co receptors in growth factor signaling.  Ref: 11668175 J Biol Chem, 2002
- We found that recombinant LAP and recombinant soluble betaglycan  [ **TGFBR3** ]  decrease the active form of TGF beta1  [ **TGFB1** ] , measured by ELISA, in a dose dependent manner in a cell free system.  Ref: 12138262 Nephron, 2002
- Betaglycan  [ **TGFBR3** ]  promoter activity was increased by RA and inhibited by the three isoforms of TGF beta  [ **TGFB1** ] .  Ref: 12399463 J Biol Chem, 2003
- We demonstrate that the expression of betaglycan  [ **TGFBR3** ]  in LLC PK1 cells results in inhibition of TGF beta  [ **TGFB1** ]  signaling as measured by reporter gene expression, thymidine incorporation, collagen production, and phosphorylation of the downstream signaling effectors Smad2 and Smad3.  Ref: 11668175 J Biol Chem, 2002
- We demonstrate instead that in LLC PK1 cells, unlike L6 cells, expression of betaglycan  [ **TGFBR3** ]  prevents association between the type I and type II TGF beta  [ **TGFB1** ]  receptors, which is required for signaling.  Ref: 11668175 J Biol Chem, 2002
- The effective dosages of LAP and soluble betaglycan  [ **TGFBR3** ]  for a 50% reduction were approximately 20 and 75 fold of the TGF beta1  [ **TGFB1** ]  concentration, respectively.  Ref: 12138262 Nephron, 2002
- We have demonstrated previously that ectopic expression of a soluble betaglycan  [ **TGFBR3** ] , also known as transforming growth factor TGF beta  [ **TGFB1** ]  type III receptor, can suppress the malignant properties of human carcinoma cells by antagonizing the tumor promoting activity of TGF beta  [ **TGFB1** ]  A.  Ref: 12183427 Cancer Res, 2002
**Neutral relationship**- In myotubes, up regulated betaglycan  [ **TGFBR3** ]  was also detectable by TGF beta  [ **TGFB1** ]  affinity labeling and immunofluorescence microscopy studies.  Ref: 12399463 J Biol Chem, 2003
- These results suggest that the ALK5 SMAD4 part of the TGFB  [ **TGFB1** ]  signalling pathway is a major target for inactivation in pancreatic carcinomas, that the expression of TGFBR2,  **TGFBR3** , and receptors involved in TGFB  [ **TGFB1** ]  activation are maintained,.  Ref: 11408925 Int J Oncol, 2001
- In comparison, the expression of betaglycan  [ **TGFBR3** ]  in L6 myoblasts enhances TGF beta  [ **TGFB1** ]  signaling, which is consistent with the published literature.  Ref: 11668175 J Biol Chem, 2002
- A third cell surface receptor, betaglycan  [ **TGFBR3** ] , serves as a co receptor for TGF beta  [ **TGFB1** ]  in some cell types, enhancing TGF beta  [ **TGFB1** ]  mediated signaling.  Ref: 11668175 J Biol Chem, 2002

**Non-interactive relationship** (e.g. studied together, co-existance, homology, etc.)

- Recombinant LAP and soluble betaglycan  [ **TGFBR3** ]  also inhibited a recombinant TGF beta1  [ **TGFB1** ]  stimulated increase in fibronectin production in MCs.  Ref: 12138262 Nephron, 2002
- These results indicate that recombinant LAP and soluble betaglycan  [ **TGFBR3** ]  suppress the glucose induced overproduction of fibronectin presumably via inhibition of TGF beta1  [ **TGFB1** ]  activity in MCs.  Ref: 12138262 Nephron, 2002
- We therefore studied the expression of UPA, UPAR, IGF2R, ALK5, TGFBR2,  **TGFBR3** , ENG, ALK1,  **TGFB1** , TGFB2, and TGFB3 in a series of 14 pancreatic carcinoma cell lines.  Ref: 11408925 Int J Oncol, 2001
- Betaglycan  [ **TGFBR3** ]  in LLC PK1 cells exhibits higher molecular weight glycosaminoglycan GAG chains than in L6 cells, and a GAG betaglycan  [ **TGFBR3** ]  mutant does not inhibit TGF beta  [ **TGFB1** ]  signaling or type I type II receptor association in LLC PK1 cells.  Ref: 11668175 J Biol Chem, 2002
- This study was performed to investigate the effects of transforming growth factor beta1  [ **TGFB1** ]  TGF beta1  [ **TGFB1** ]  latency associated peptide LAP and betaglycan  [ **TGFBR3** ]  on TGF beta1  [ **TGFB1** ]  activity, and on the glucose induced overproduction of fibronectin in cultured human mesangial cells MCs.  Ref: 12138262 Nephron, 2002
- 0.01 reduced by the addition of 10 nmol l LAP and 10 nmol l soluble betaglycan  [ **TGFBR3** ]  with no significant change in total active latent TGF beta1  [ **TGFB1** ] .  Ref: 12138262 Nephron, 2002
- Forced expression of betaglycan  [ **TGFBR3** ]  in CC myoblasts increases their responsiveness to TGF beta2, suggesting that it performs a TGF beta  [ **TGFB1** ]  presentation function in this cell lineage.  Ref: 12399463 J Biol Chem, 2003
- Transforming growth factor beta 1  [ **TGFB1** ]  latency associated peptide and soluble betaglycan  [ **TGFBR3** ]  prevent a glucose induced increase in fibronectin production in cultured human mesangial cells.  Ref: 12138262 Nephron, 2002
